# Supplementary material for: The Role of Neuropeptide Y in Adipocyte-Macrophage Crosstalk during High Fat Diet-Induced Adipose Inflammation and Liver Steatosis
Source: Biomedicines. 2021 Nov 22;9(11):1739. doi: 10.3390/biomedicines9111739 (PMC8615496; doi:10.3390/biomedicines9111739)

Figure S2A Western blot in iWAT
(W, wild type; K, knockout, C, CRF1; H, HFD; P, positive control)

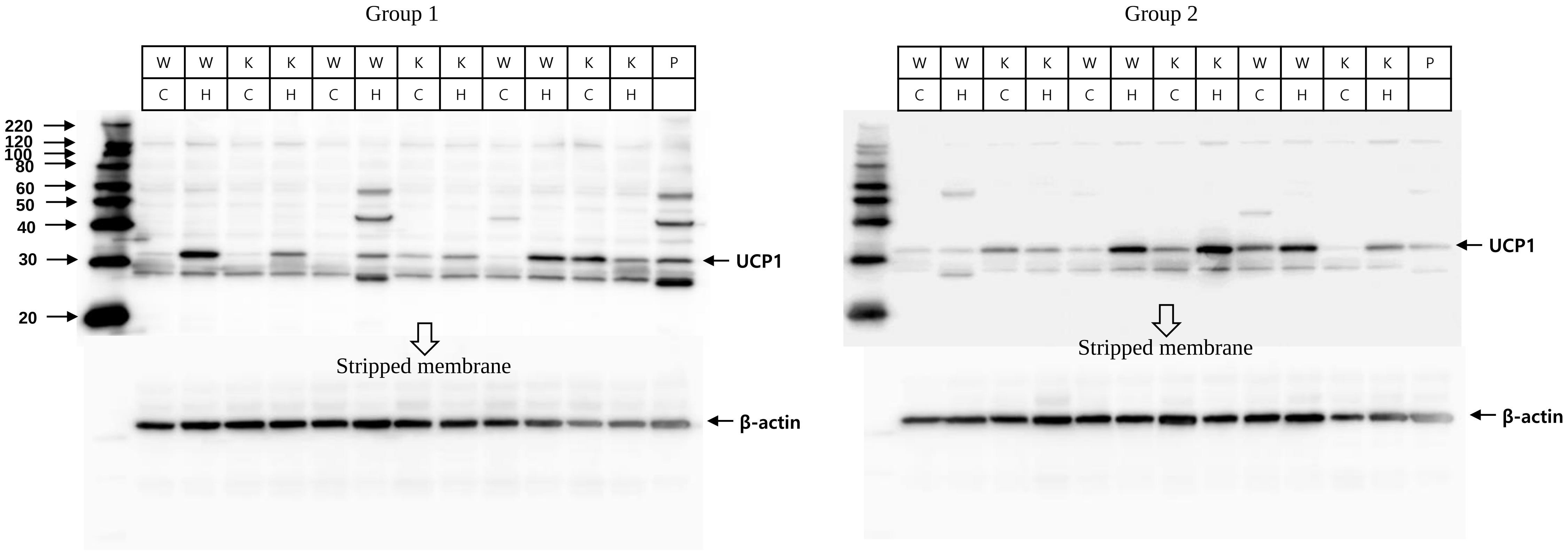

Figure S2A Western blot in BAT
(W, wild type; K, knockout, C, CRF1; H, HFD; P, positive control)

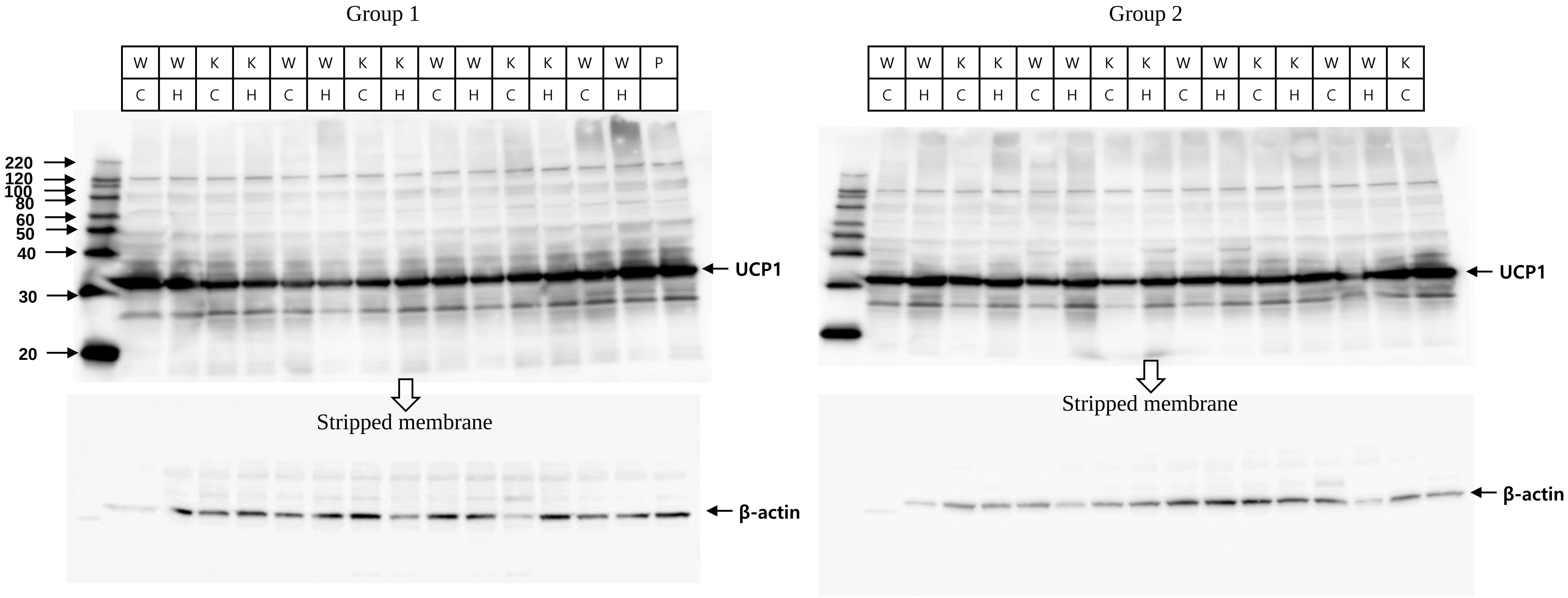

Supplement: Supplementary file 1 [file biomedicines-09-01739-s001.zip › Figure S2A (Blot results).pdf]
