# Supplementary material for: The Role of Neuropeptide Y in Adipocyte-Macrophage Crosstalk during High Fat Diet-Induced Adipose Inflammation and Liver Steatosis
Source: Biomedicines. 2021 Nov 22;9(11):1739. doi: 10.3390/biomedicines9111739 (PMC8615496; doi:10.3390/biomedicines9111739)
Supplement: Supplementary file 1 [file biomedicines-09-01739-s001.zip › S1-S4.pdf]

Figure S1.

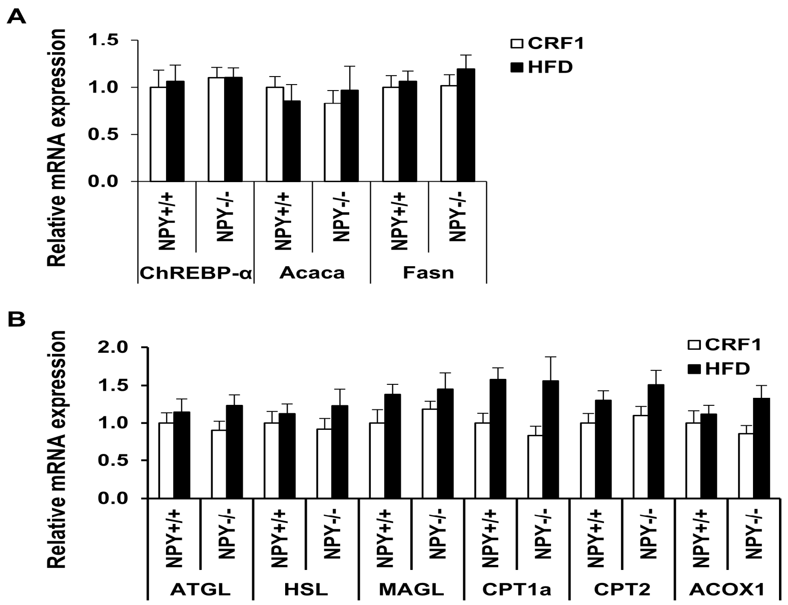

Figure S1. NPY deficiency does not affect the mRNA expression of genes involved in (A) de novo lipogenesis, (B) lipolysis and fatty acid oxidation in eWAT from WT and KO mice. All data are presented as the mean ± SEM (n = 5-6 per group).

Figure S2.

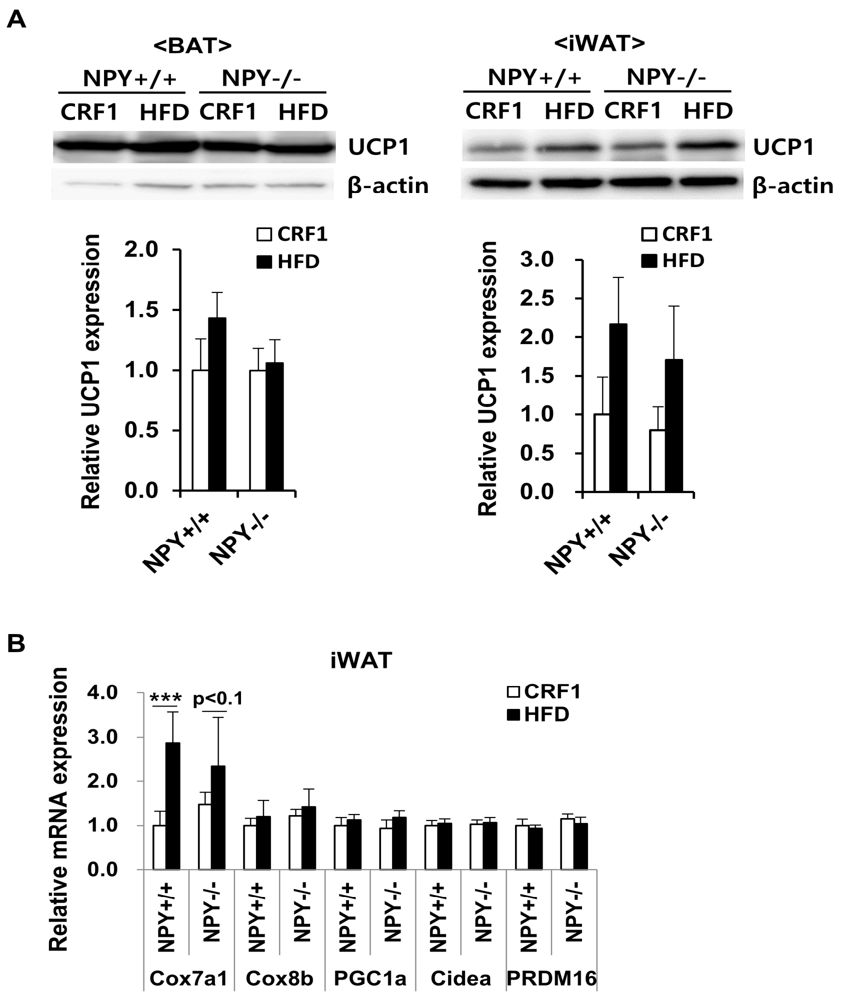

Figure S2. NPY deficiency does not affect thermogenesis in BAT and iWAT from WT and KO mice. (A) Western blotting for Ucp1 in BAT and iWAT from $NPY^{+/+}$ and $NPY^{-/-}$ mice (top panel) and quantified (bottom panel), (B) mRNA expression level of thermogenesis-related genes in iWAT from WT and KO mice. All data are presented as the mean ± SEM (n = 5-6 per group), Student's two-tailed *t* test, $^{***}p < 0.001$.

Figure S3.

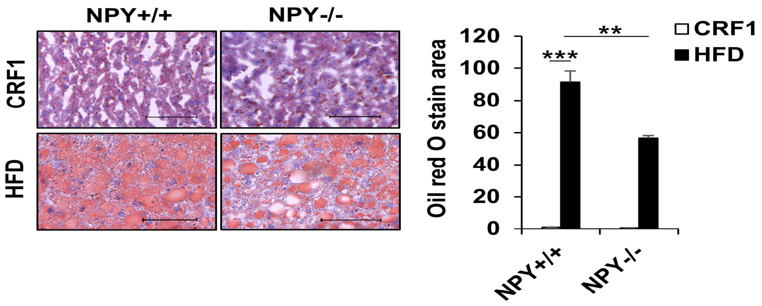

Figure S3. NPY deficiency improves HFD-induced liver steatosis, Oil red O staining in liver from $NPY^{+/+}$ and $NPY^{-/-}$ mice. Scale bar, 100 μm. All data are presented as the mean ± SEM (n = 3 per group), Student's two-tailed *t* test, $^{**}p < 0.01$ and $^{***}p < 0.001$.

Figure S4.

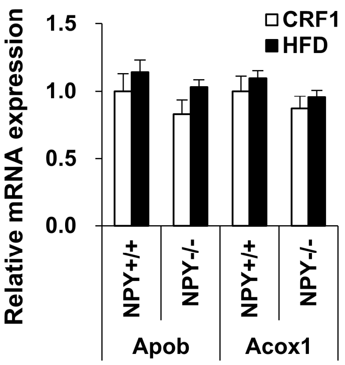

Figure S4. NPY deficiency does not affect mRNA expression of genes involved in secretion of very low density lipoprotein (Apob) and beta-oxidation (Acox1) in liver from WT and KO mice. All data are presented as the mean ± SEM (n = 5-6 per group).
