# Supplementary material for: The Role of Neuropeptide Y in Adipocyte-Macrophage Crosstalk during High Fat Diet-Induced Adipose Inflammation and Liver Steatosis
Source: Biomedicines. 2021 Nov 22;9(11):1739. doi: 10.3390/biomedicines9111739 (PMC8615496; doi:10.3390/biomedicines9111739)
Supplement: Supplementary file 1 [file biomedicines-09-01739-s001.zip › supplemental information.pdf]

Supplementary information

Materials and methods

1. Western blotting

Antibodies for UCP1 was obtained from Abcam (Cambridge, UK), and Anti-β-actin was purchased from Cell Signaling Technology (Danvers, MA, USA). Enhanced chemiluminescence (ECL) western blotting detection reagents and ECL-anti-rabbit or mouse IgG, horseradish peroxidase-linked species-specific whole antibodies were purchased from Amersham Pharmacia Biotech (Little Chalfont, UK). All samples were boiled for 3 min and chilled on ice. Proteins were separated by SDS-PAGE and transferred to a nitrocellulose membrane. Membrane were immediately placed in blocking solution (2% bovine serum albumin (BSA) or 5% non-fat dry milk in Tris-buffered saline with 0.1% Tween 20 (TBS-T) buffer) for 1 h and incubated with the primary antibody for overnight at 4°C followed by the secondly antibody for 1 h at room temperature. Antibody labeling was detected using ECL as per the manufacturer's instructions. Specific signals were quantified by Fluorchem (DE500-5T, Alphainnotech Corporation, San Leandro, CA) with associated image analysis software, AlphaEase FC.

2. Oil red O staining in tissue

Frozen liver samples were embedded in OCT and cut in 10 µm section. Sections were fixed with 10% formalin for 5 min, washed with 60% isopropanol, then saturated with Oil Red O (1% w/v, 60% isopropanol) for 10 min, washed in 60% isopropanol and rinsed in distilled water. The sections were mounted in mounting medium with coverslips. Images were acquired on a BZ-X700 (Keyence, Osaka, Japan). The Oil red O staining area was measured by an application of Image J (Analyzed area: 0.2 mm^2).

Figure Legends of supplementary data

Supplementary Figure S1. NPY deficiency does not affect the mRNA expression of genes involved in (A) de novo lipogenesis, (B) lipolysis and fatty acid oxidation in eWAT from WT and KO mice. All data are presented as the mean ± SEM (n = 5-6 per group).

Supplementary Figure S2. NPY deficiency does not affect thermogenesis in BAT and iWAT from WT and KO mice. (A) Western blotting for Ucp1 in BAT and iWAT from $NPY^{+/+}$ and $NPY^{-/-}$ mice (top panel) and quantified (bottom panel), (B) mRNA expression level of thermogenesis-related genes in iWAT from WT and KO mice. All data are presented as the mean ± SEM (n = 5-6 per group), Student's two-tailed *t* test, $^{***}p < 0.001$.

Supplementary Figure S3. NPY deficiency improves HFD-induced liver steatosis, Oil red O staining in liver from $NPY^{+/+}$ and $NPY^{-/-}$ mice. Scale bar, 100 µm. All data are presented as the mean ± SEM (n = 3 per group), Student's two-tailed *t* test, $^{**}p < 0.01$ and $^{***}p < 0.001$.

Supplementary Figure S4. NPY deficiency does not affect mRNA expression of genes involved in secretion of very low density lipoprotein (Apob) and beta-oxidation (Acox1) in liver from WT and KO mice. All data are presented as the mean ± SEM (n = 5-6 per group).
