# Supplementary material for: The Role of Neuropeptide Y in Adipocyte-Macrophage Crosstalk during High Fat Diet-Induced Adipose Inflammation and Liver Steatosis
Source: Biomedicines. 2021 Nov 22;9(11):1739. doi: 10.3390/biomedicines9111739 (PMC8615496; doi:10.3390/biomedicines9111739)
Supplement: Supplementary file 1 [file biomedicines-09-01739-s001.zip › Table S1.pdf]

Table S1. Gene name, symbols, and primer sequences.

Gene Name	Gene Symbol	Forward Primer	Reverse Primer
**18s ribosomal RNA**	18S	TTCTGGCCAACGGTCTAGACAAC	CCAGTGGTCTTGGTGTGCTGA
**Acetyl-Coenzyme A carboxylase alpha**	Acaca	TGGCAGACCACTATGTTCCA	GTTCTGGGAGTTTCGGGTTC
**Acyl-CoA oxidase 1**	Acox1	TTGGAAACCACTGCCACATA	GCCAGGACTATCGCATGATT
**Apolipoprotein B**	Apob	TTGGCAAACTGCATAGCATCC	TCAAATTGGGACTCTCCTTTAGC
**Adipose triglyceride lipase**	Atgl	ACACCAGCATCCAGTTCAA	GGTTCAGTAGGCCATTCCTC
**Chemokine ligand 2**	Ccl2	TTAAAAACCTGGATCGGAACCAA	GCATTAGCTTCAGATTTACGGGT
**CD36 molecule**	Cd36	TCCTCTGACATTTGCAGGTCTATC	AAAGGCATTGGCTGGAAGAA
**CD68 antigen**	Cd68	GCCCGAGTACAGTCTACCTGG	AGAGATGAATTCTGCGCCAT
**Carbohydrate responsive element binding protein alpha**	ChREBP-α	CGACACTCACCCACCTCTTC	TGTTCAGCCGGATCTTGTC
**Cell death-inducing DFFA-like factor alpha**	Cidea	ACAGAAATGGACACCGGGTAGT	CGAAGGTGACTCTGGCTATTCC
**Cytochrome C oxidase subunit 7a polypeptide 1**	Cox7a1	CAGCGTCATGGTCAGTCTGT	AGAAAACCGTGTGGCAGAGA
**Cytochrome C oxidase subunit 8b polypeptide**	Cox8b	GCGAAGTTCACAGTGGTTCC	GAACCATGAAGCCAACGACT
**Carnitine palmitoyltransferase 1a**	Cpt1a	GTCAAGCCAGACGAAGAACA	CGAGAAGACCTTGACCAATAG
**Carnitine palmitoyltransferase 2**	Cpt2	CAGTTCAGGAAGACAGAAGTG	CGAAGTGTCTTCAGAAACCG
**Cytochrome P450, family 4, subfamily a, polypeptide 14**	Cyp4a14	TTCTTTCGCCTGCGGAATGC	CACTCCATCTGTGTGCTCGTGA
**Diacylglycerol O-acyltransferase 2**	Dgat2	CCAGCACTACTTTGGCATAATGC	CCTCTAGGTATGTCTGATGCAGC
**Fatty acid synthase**	Fasn	AAGGCTGGGCTCTATGGATT	GGAGTGAGGCTGGGTTGATA
**Fatty acid transporter protein 2**	Fatp2	GATGCCGTGTCCGTCTTTTAC	GACTTCAGACCTCCACGACTC
**Fatty acid transporter protein 5**	Fatp5	CCAGTGTGCTGATTGTGGAT	GGCCAAGGTAGAAGCAGTGA
**glyceraldehyde-3-phosphate dehydrogenase**	Gapdh	ACTTTGTCAAGCTCATTTCC	TGCAGCGAACTTTATTGATG
**Interleukin 6**	Il6	TAGTCCTTCCTACCCCAATTTCC	TTGGTCCTTAGCCACTCCTTC
**Lipase, hormone sensitive**	Lipe	TCCTGGAACTAAGTGGACGCAAG	CAGACACACTCCTGCGCATAGAC
**Lipoprotein lipase**	Lpl	CTGCTGGCGTAGCAGGAAGT	GCTGGAAAGTGCCTCCATTG
**Monoglyceride lipase**	Magl	TTTCTGGCATGGTCCTGATT	ATTGAGCAGTTTGGCAGCA

**Neuropeptide Y**	Npy	TATCTCTGCTCGTGTGTTTG	GTTCTGGGGGCATTTTCTG
**Neurpeptide Y receptor Y1**	Npy1r	GTCCTTGCAGTGGCTTCTTC	TGATTCGCTTGGTCTCACTG
**Peroxisome proliferator-activated receptor gamma coactivator 1 alpha**	Pgc1α	ATGTGTCGCCTTCTTGCTCT	CGGTGTCTGTAGTGGCTTGA
**Perilipin 1**	Plin1	AGATCCCGGCTCTTCAATACC	AGAACCTTGTCAGAGGTGCTT
**Peroxisome proliferator-activated receptor gamma 2**	Pparγ2	TGGGTGAAACTCTGGGAGAT	GCTGGAGAAATCAACTGTGG
**PR domain containing 16**	Prdm16	TCTTACTTCTCCGAGATCCGAAA	GATCTCAGGCCGTTTGTCCAT
**Tumor necrosis factor alpha**	Tnf-α	CCCTCACACTCAGATCATCTTCT	GCTACGACGTGGGCTACAG
**Uncoupling protein 1**	Ucp1	CACTCAGGATTGGCCTCTACGAC	GCTCTGGGCTTGCATTCTGAC
